# The Epidemic of Neonatal Abstinence Syndrome, Historical References of Its’ Origins, Assessment, and Management

**DOI:** 10.3389/fped.2018.00033

**Published:** 2018-02-22

**Authors:** Enrique Gomez-Pomar, Loretta P. Finnegan

**Affiliations:** ^1^Division of Neonatology, Department of Pediatrics, University of Kentucky, Lexington, KY, United States; ^2^The College on Problems of Drug Dependence, Inc., Philadelphia, PA, United States

**Keywords:** drug withdrawal, neonatal abstinence syndrome, neonate, opioid, methadone, buprenorphine

## Abstract

Neonatal abstinence syndrome (NAS) refers to a constellation of signs that are present in some newborn infants resulting from the abrupt cessation of passive transfer of maternal opioids used during pregnancy. The classic NAS refers to infants born to mothers who used opioids during pregnancy, but the term has broadened to include infants whose mothers have used or abused other psychoactive substances during pregnancy that contribute to the expression of the syndrome. Pregnant women who use opioids do so illicitly, and/or as medically prescribed for pain relief, and/or as medication assisted treatment for opioid dependence. The first case of NAS in infants and the subsequent treatment (or lack thereof) was reported in 1875 and was called Congenital Morphinism. By 2012, the incidence of NAS increased to more than 30 per 1,000 hospital live births, along with an increase in the number of infants being treated pharmacologically for NAS, resulting in an increase in the length of stay and healthcare expenses. We present historical references on NAS, the various factors and events that led to its increasing prevalence and today’s current epidemic. We also review the current tools to assess infants with NAS and treatment options in its management.

“The one who does not remember history is bound to live through it again.” [Georges Santayana, Spain (1863–1952)]

## The Early Days of *In Utero* Exposure to Opioids

The first description of opium use goes back 6,000 years; then known as “the plant of joy” (1) being first isolated by Sumerians around 2,000 years BC (2). Initially used to cause euphoria (probably as part of religious rituals), it was found that it could also be used to help people die painlessly and to calm crying children (1, 2). Medical use of opium included treatment of a variety of illnesses such as: pain (headaches, menstrual cramps, etc.), respiratory (bronchitis, asthma, cough), infections (fever, leprosy), and mental problems (melancholy) (1). As opium use spread throughout Europe, China and India, its abuse and addiction appeared and advanced but it wasn’t until 1803, after the isolation of its main component, morphine, and the expansion of its medical use, that addiction became a public health problem (1, 2).

Morphine was marketed by Merck & Co., Inc. in 1827 to be used for pain relief and alcoholism treatment (3). In 1853, morphine became the first drug to be used intravenously for pain control during minor surgeries (1, 2). However, morphine was found to be as addictive as opium, and efforts were made to synthetize an opium derivative that did not cause addiction. The assumption was that if potency was high, less quantity should be needed resulting in less addiction (2, 3). This effort resulted in the development of heroin by Heinrich Dreser in 1898, a drug five times more potent than morphine (1, 2). Heroin was distributed by Bayer (2) AG (1898–1910) as an over-the-counter medication to treat colds, sore throats, pneumonia, and tuberculosis (1). Secondary to the belief that heroin was not addictive, it was also proposed as treatment for morphine-addicted patients.

The introduction of opium in the US started between 1850 and 1870 with the arrival of Chinese citizens during the California Gold Rush (1–3). Its use rapidly extended to all American major cities (1). From 1900 to 1924, heroin was distributed in the US as an over-the-counter medication for the treatment of colds and the flu and was marketed as safe to be given to pregnant women and infants (1). Opium was initially consumed using smoking devices, but because of the poor reputation of smokers (most likely Chinese laborers and white criminals), consumption spread to the middle and upper classes mostly after the availability of oral and intravenous forms (4). There were about 300,000 opioid addicts at the beginning of the twentieth century, and two thirds of those consumers were women who started using prescription opioids for a variety of illnesses and continued using them afterward (3, 4). Opiate abuse was also exacerbated by poor governmental control regarding the sale and distribution of narcotics (4).

At first, public perception of opium addicts was much positive than that of alcoholics, influencing the widespread use of morphine and heroin (4). However, these attitudes started to change at the beginning of the twentieth century, as negative perceptions of who addicts were, and how they became addicted, appeared (4). Concomitantly, physicians became more aware of the addictive effects of morphine and heroin and its deleterious effects on their patients, which resulted in fewer new cases of medical addiction (4–7).

The first attempt to regulate opiates in the US came in 1875, by the city of San Francisco, who enacted the Opium Den Ordinance, which declared public smoking (opium dens) illegal (1). Around that time, alarmed physicians raised their voices to remind their colleges about the dangers of opiate addiction and to challenge the proposed standards of care that used heroin for many afflictions including the treatment of addicts (8). This prompted the establishment of more controls across the states (7). By 1914, the federal government passed the Harrison Narcotic Act (4, 7) which required anyone who imported, produced, sold or dispensed “narcotics” to register and keep records of their operations. The Act also allowed authorities to prosecute unregistered distributors. The Harrison Act was later replaced by the 1970 Controlled Substances Act (7) which has been enforced by the Drug Enforcement Administration since its creation in 1973.

The term opioid was introduced in the late 1950s to refer to the synthetic narcotics (1). Oxycodone is a semisynthetic opioid synthesized from thebaine, an opioid alkaloid found in the Persian poppy, and one of the many alkaloids found in the opium poppy (9). Oxycodone was initially synthetized in 1916 in Germany and used for pain control, especially during World War II. In the post war time, and after the negative image of heroin, oxycodone was introduced in the US. It has since become one of the best selling prescription drugs in the country (1). By 1939, meperidine, the first synthetic opioid, was created; this was followed by the synthesis of methadone in 1946 (2, 10). Due to a nationwide effort to create treatment programs for opiate addiction, methadone was first used as a maintenance medication in the 1950s (10). Buprenorphine was discovered in 1966 and found to be beneficial for the treatment of opioid dependence as well as methadone (10, 11). Buprenorphine use was approved by the Food and Drug Administration in 1985. However, physicians were not permitted to prescribe buprenorphine in treatment settings other than Opioid Treatment Programs until the Drug Addiction Treatment Act of 2000 (part of the Children’s Health Act of 2000) (10–12).

The use of opioids for pain control in terminally ill patients started in 1948 after Cicely Saunders founded the Hospice Care movement in London (13). Ms. Saunders introduced the concept of a pain-free dignified death using opioids to prevent pain instead of using them as treatment. This movement was adopted by the US and, in 1984, the Compassionate Pain Relief Act was passed allowing physicians to legally treat terminally ill patients with heroin (1). However, in 1986 a new call was made to use chronic opioid treatment for non-malignant pain, based on the incorrect assumption that opioids can be used chronically without causing serious addiction (14, 15).

## Inadequate Prescribing Practices Leading to a National Epidemic

Opioid prescriptions shifted to the treatment of chronic pain resulting in a steep increase in the abuse of prescribed opioids with a reported 259 million prescriptions for opioid medications in 2012 (16, 17). Approximately half of all emergency room visits related to drug misuse in 2011 are related to nonmedical use of prescription drugs, 40% of these visits related to opioid pain relievers (18). Therefore, treatment admissions have more than quadrupled between 2002 and 2012, along with deaths related to overdose (11). In 2014, there were almost 19,000 deaths related to the overdose of prescription opioids (19). As of 2015, there are an estimated two million people living in the US with a substance abuse disorder related to prescribed opioids (11).

However, due to stricter controls on prescribed opioids (18), there has been a shift to heroin and other options easier to find (11, 20). Evidence also shows that individuals who abuse opioids orally, will eventually switch to injection routes thus resulting in additional health risks related to the injections (20–23). Admissions for prescribed opioid abuse decreased from 2004 to 2013, meanwhile, there was an increase in the number of individuals using injection and smoking/inhalation methods (21). In 2015, there were an estimated 591,000 people with heroin use disorder and approximately 13,000 deaths related to overdose (11); more than five times the number in 2002 (22).

Children and adolescents have not escaped this epidemic; from 1997 to 2012 more than 22,000 children were treated for opioid poisoning (16). Those among the ages 0–5 years experienced the largest number of exposures to opioids, mostly from unintentional sources (accidental ingestion or therapeutic error) (16). However, among teenagers the majority of opioid exposure was intentional, being more significant among boys (16). A survey from 2015 found that 1 in 23 high school seniors reported misusing opiates (18) and research has also shown that teens and adolescents who abuse prescription medications are also more likely to use other drugs (18).

Sex differences related to substance abuse, like cause of consumption and drug metabolism, make women a unique group (24). As described earlier, opioid consumption was already common among women during the nineteenth century (4), possibly relating to women being more likely to suffer from chronic pain and being more susceptible to craving and relapse (24). Drug abuse and deaths due to overdose are more common in men; however, the rate of overdose is increasing more sharply in women compared to men (18). Misuse of prescription drugs is also more common among men, except in adolescents; adolescent girls, age 12–17, surpass boys in the use of nonmedical prescription drugs (18). Deaths related to overdose of prescription pain relievers are also increased more rapidly in women compared to men; reaching an overwhelming 18 women dying per day due to overdose in 2010 (24).

Opioids use during pregnancy results in complications to the newborn known as neonatal abstinence syndrome (NAS) (25–29). From 1995 to 2009, opioid prescriptions for pain management in pregnant women doubled (11). In a cohort of more than 100,000 pregnant women exposed to opioids, nearly 96% were non-maintenance prescriptions (30). Women who are pregnant or have young children may not look for addiction treatments or drop off from treatment due to the fear of legal repercussions or that they will be seen as unable to take care of their children (12, 24, 31). Stopping opioids during pregnancy is not an option as it is associated with poor neonatal outcomes and may result in fetal demise (11, 31).

Currently, there is no federally approved medication treatment for pregnant women with an opioid disorder; however, methadone and buprenorphine (category C drugs as per the Food and Drug Administration) appear to be effective and safe options during pregnancy (24, 31). Still, knowledge gaps persist concerning methadone pharmacodynamics, pharmacokinetics and the lack of guidelines for adequate dosing during pregnancy (12).

## Neonatal Abstinence Syndrome

Prior to 1875, infants born to women who were opioid dependent were thought to be unaffected because morphine use among women was associated with sterility and loss of sexual desire (32). However, in 1875 several cases of deceased infants born from mothers dependent on morphine were reported (3, 33, 34). This condition was called Congenital Morphinism and it was described in full term infants who appeared normal at birth then began crying inconsolably on the third day of life; some of these infants were reported to develop generalized seizures (32, 33). Due to a lack of knowledge of the cause of these signs, NAS was frequently fatal to newborns (3, 33, 34). As early as 1901, it was recognized that this was the result of the infant withdrawing from the cessation of the passive transfer of maternal morphine and that providing the infant with medication would ease his/her signs (33, 34). Later on, infants were given opium in small quantities to treat their symptoms with reports of success (34).

Due to the low-molecular-weight and lipid solubility of opioids, they pass freely through the placenta and to the infant (25–27). Fetuses of mothers using opioids and receiving no or inadequate prenatal care have an increased risk of having preterm birth, intrauterine growth restriction, and an increase incidence of fetal demise (26, 31, 35, 36). Several drugs have been identified to cause some signs of withdrawal in the infant, but the most common cause is *in utero* opioid exposure (3, 25–27, 37–40). The cutting of the umbilical cord causes an abrupt termination of the supply of opioids to the infant and increases the risk of developing NAS (25–27).

Clinically recognizable abstinence signs appear in 60–80% of opioid-exposed neonates. Their spectrum of signs is affected by variables such as the total fetal exposure, the amount and purity of the drugs taken by the mother, length of use, maternal and infant drug metabolism, and the individual kinetics of placental drug transfer (25–27, 38, 40). Genetic and epigenetic factors play an important role in the incidence and severity of NAS in neonates (41, 42). Timing of presentation of NAS is usually within the first 72 h of life but it can occur as late as 7 days of life (27, 38). Signs of NAS can be classified in: (a) neurologic manifestations due to increased excitability, including tremors, excessive and/or high pitch cry, hyperactive Moro, increased muscle tone, seizures; (b) gastrointestinal manifestations include diarrhea, vomiting, uncoordinated sucking, and swallowing; and (c) autonomic manifestations include fever, sweating, nasal stuffiness, and increased respiratory rate (25, 27, 37, 40, 43, 44). The most common signs are related to the neurologic and gastrointestinal manifestations, however, there is variability in the presentation of signs among neonates (27, 38, 40, 45).

Opioid excretion in breastmilk was recognized as early as 1901 (34). Breastfeeding was encouraged in these infants as it helped “calm them” and in some occasions ease their symptoms without pharmacological intervention (34, 46). Evidence has shown that mothers on methadone or buprenorphine have low, safe levels of the medication in their breast milk (47). Therefore, the current recommendation is to encourage breastfeeding in those infants whose mothers are on a supervised opioid maintenance program, are compliant and not using street drugs (27). Breastfeeding of these infants has demonstrated to be beneficial in decreasing their NAS signs and hospital stay and improving maternal–infant attachment most probably due to the soothing effect of breastfeeding (27, 47, 48).

## An Objective Evaluation of Infants with NAS

Assessment and diagnosis of NAS starts with clinical suspicion based on maternal history (27, 32, 49). In order to provide an objective way to identify and categorize infants with NAS, several scoring systems were proposed (50), including the Finnegan Neonatal Abstinence Scoring System (FNASS) (25, 37), the Lipsitz tool (51), the Neonatal Narcotic Withdrawal Index (52), and the Ostrea tool (35). Currently, the FNASS is the most commonly used scale (25, 27, 37, 53–56).

The FNASS was developed in 1975 using a clinimetric approach based on the most common signs of infants with NAS (25, 37, 57). The FNASS consists of 21 items, was analyzed for interuser reliability [mean interrater reliability coefficient of 0.82 (0.75–0.96)] and validated for the diagnosis of NAS (26, 58). The FNASS provides cutoff points (3 continuous scores ≥ 8 or 2 continuous scores ≥ 12) for the identification of the infants that may require pharmacological treatment and pharmacological guidelines for the treatment of infants with NAS (25, 37, 59). Subsequent analysis of the external factors that may influence the FNASS showed that it is an objective and reliable tool for the diagnoses and management of infants with NAS (60).

The Lipsitz tool was developed around the same time of the FNASS (1975) (51). It contains 11 items with scores from 0 to 3 and proposes to evaluate infants twice per day 90 min prior to feeds. It found sensitivity of 77% using scores ≥ 4 as an indication of withdrawal signs. Other scales like the Neonatal Narcotic Withdrawal Index (7 category scale that proposes a cutoff of 5 obtained twice during a 24-h period) (52) and the Ostrea tool (a six criteria scale) (35) were proposed; however, these scales did not gain much popularity in clinical settings.

The FNASS is a comprehensive and lengthy tool so there have been several attempts to modify it (40). The Neonatal Withdrawal Inventory (61) proposed an 8–point checklist that was derived from the FNASS. The investigators reported on inter-rater reliability, sensitivity and specificity using the same cutoff points as the FNASS; however, the scoring system was not validated. The FNASS-short form developed by Maguire et al (62) proposed a 7-point checklist using the same cutoff value as the FNASS. The simplified FNASS (sFNASS) is a 10-point scale that was statistically derived using the FNASS scores of a database of 185 patients (63). This was subsequently validated with a different database of 182 infants from another NICU with excellent correlation to the original FNASS. The sFNASS proposes cutoff values of 6 and 10 to identify scores of 8 and 12 on the original FNASS. However, a prospective evaluation of the sFNASS is needed (63).

The Maternal Opioid Treatment: Human Experimental Research (MOTHER) project developed a score by modifying the FNASS (64). The MOTHER NAS scale is a 19 item scoring system developed by adding (failure to thrive and irritability), removing (myoclonic jerks, mottling, nasal flaring and excessive sucking), or modifying the scores in the items of the original FNASS (65). Infants were evaluated twice daily and pharmacological treatment was offered by their proposed guidelines (64). Short screening tools based on the MOTHER NAS scale have also been proposed (66); however, its validation awaits further studies.

At present, there is no national consensus as to which tool to use, the cutoff points for treatment, and the interval between assessments (27, 50, 53, 56). A 2006 review by Sarkar et al. found that the FNASS was used by 65% of the 75 units that were analyzed, which is consistent with other reports (25, 27, 37, 53–56, 67). Conversely, most of the concerns were based in the length of the tool and the fact that it was designed for opioid withdrawal not for polydrug users. However, the majority of signs that infants experience from *in utero* polydrug exposure concomitantly with opioids (benzodiazepines, SSRI, sedatives) are similar and additive to those seen with opioids alone (27).

An evaluation of the validity of the cutoff points was made by Zimmermann et al. in 2010 (68). They applied the FNASS to infants without a history of opioid exposure and found that scores ≥8 should be considered pathological (68). Infants without opioid exposure commonly have scores <8 and, if a score of 8 is found in one evaluation, they subsequently became normal (68). This finding validates the suggestion of requiring 3 continuous abnormal scores to consider pharmacological treatment. To consider, in the development of the FNASS, 200 babies born without drug exposure (including no analgesics for labor and delivery) never received a score of ≥8 or higher. Most were in the 1–5 range. Current recommendations encourage each nursery to develop a protocol that includes a standard assessment tool for infants with NAS (27, 32).

## Management of NAS

Management of infants suffering from NAS has two main components, non-pharmacological and pharmacological interventions that are used as needed. Non-pharmacological options have been used since the early identification of congenital morphinism (34, 46). The main components of a non-pharmacological approach are recommended to decrease the environmental stimuli that the infant is exposed to (minimize environmental stimuli, swaddling, rocking) and minimize hunger (demand feedings) (32, 69–71). These interventions have demonstrated a decrease in the severity of symptoms (32, 50, 69) and new evidence is showing that they may be a key component in the management of infants with NAS (67, 69).

Recently, a new approach called the Eat, Sleep, Console (ESC) model was reported (69). This model is based on intensive non-pharmacological therapy of infants with NAS, specifically those from mothers on methadone. Over a period of 8 years, the investigators showed a decreased need for pharmacological therapy with a shortened hospital stay with the ESC method (when comparing a historical comparison group to the post intervention group) (69). However, it seems that constant maternal rooming-in is key to the model, making it difficult to apply nationwide and the model is based on a subjective evaluation (without a report of other signs of NAS), making it difficult to replicate (69).

Another concern is at which point the non-pharmacological interventions alone will have deleterious effects on the neurological development of infants with NAS. Infants with antenatal opiate exposure will have an increased noradrenergic activity, among other neurological abnormalities, once they are born and the opiate supply ceases (32, 43, 72, 73). Therefore, we question that not offering pharmacological treatment is enough for these infants and could be detrimental in the long term. Further research is necessary to evaluate the long term effects of the ESC model.

At present, infants diagnosed with NAS that are in need of a pharmacological treatment are managed in a neonatal intensive care unit (NICU) (28, 74). An infant with NAS admitted to the NICU will have difficult access to rooming-in and a low stimuli environment. Holmes et al. reported a program in which 207 infants were treated in a pediatric unit instead of a NICU (74). This allowed constant rooming-in and resulted in decreased of use of pharmacological treatments, length of stay and overall health cost in an overall safe environment to the infant (74).

Initially, when NAS (Congenital Morphinism) was identified, no pharmacological treatment was given and these infants died, not only from the lack of treatment for NAS but, in some, from prematurity (3, 33, 34). Once the condition was recognized to be caused by the interruption of the placental supply of opiates, pharmacological interventions were provided resulting in improvement of the survival rates (33, 34). Initially, infants were treated with opium, paregoric and oral diluted morphine (34). Currently, the most common medications used in US nurseries include morphine and methadone with phenobarbital, clonidine, and buprenorphine being used alone or as adjuvant therapy. However, pharmacological management is not standardized; therefore, medication dosing and weaning varies center to center (27, 53, 55, 56, 75, 76). The threshold as to when an infant needs pharmacological intervention is questioned by some clinicians and, if treated, the choice of which medication to use remains controversial (27, 50, 53, 56, 67, 77–79).

Morphine and methadone have been the drugs of choice to treat infants where maternal opioid exposure is demonstrated and NAS is established (50, 53, 64). Morphine is given orally, typically every 3–4 h at 0.05–0.2 mg/kg/dose (50, 80). If the infant does not improve with the initial dose of medication, it can be increased to obtain the desired effect. Morphine can be weaned every 24–48 h (50, 80).

Methadone is also given orally every 4–12 h, titrated in a range of 0.05–0.1 mg/kg/dose, and then weaned over time (50). However, the use of methadone is controversial due to its long half-life and prolonged excretion rate that could require longer hospitalization (65, 79).

Clonidine, a non-opioid α2-adrenergic receptor agonist, has been recommended as a safe alternative for single-drug therapy of infants with NAS (72). Clonidine eases the signs and does not include the narcotic effects of opioids (80). Clonidine can be initially started at 0.5–1 μg/kg, followed by 0.5–1.25 μg/kg per dose q 3 h with proposed increments of 25% of the initial dose and weaning by 10% of the maximum dose every 48 h (50). A potential side effect is blood pressure fluctuations; however, no studies have reported this side effect at the doses used for the treatment of infants with NAS (72, 80). Clonidine also can be used as adjuvant medication when opioids were initially used.

Phenobarbital, a γ-amino butyric acid agonist with sedative and anticonvulsant properties, has been used for years for the treatment of NAS (70). However, phenobarbital is most commonly used as an adjuvant therapy and not as a single-drug medication (32, 80). Phenobarbital requires a loading dose of 5 mg/kg IV, IM, or PO and a maintenance dose of 3–5 mg/kg divided into three doses (every 8 h). Another approach is a loading dose of 20 mg/kg with a maintenance dose of 2–6 mg/kg day to achieve plasma level concentrations between 20 and 30 μg/mL (80, 81). Once the infant is controlled, phenobarbital can be weaned by 15% of the daily dose until the medication is discontinued (80).

Treatment of NAS must take into consideration factors that have been shown to enhance the expression of NAS and effect the response of infants to non-pharmacological and pharmacological interventions. These include neonatal (41, 42) (gestational age, neonatal metabolism, genetic predisposition, and epigenetics), maternal (12, 30, 38) (smoking, type, length, quality, and quantity of the used drug, SSRI use and the enrollment in Medication Assisted Therapy), and external factors (69) (decision to breastfeed and rooming-in possibility). Significant variability still persists regarding pharmacological treatment of infants with NAS (dose initiation, increments, and weaning). Therefore, each unit needs to develop a protocol to provide consistent treatment to the affected infants (27, 50).

## NAS as a National Epidemic

The incidence of NAS has steadily increased since the 1970s and has now become a significant public health problem (30, 37). NICU admissions due to NAS have increased from 7 cases/1,000 admissions in 2004 to 27 cases/1,000 admissions in 2013 with an increase in the median length of stay for infants with NAS from 13 to 19 days (28). The proportion of infants who received pharmacotherapy also increased, 74% in 2004–2005 to 87% in 2012–2013, resulting in a 35% increase in hospital costs (28).

Prescribed opioids are prevalent among mothers of infants with NAS. A cohort of more than 10,000 infants with NAS showed that methadone, opioid pain relievers and buprenorphine was used in 31, 24, and 15% of the mothers, respectively (28). Pregnant women tend to use less illicit drugs and smoking compared to non-pregnant women, excluding the group age between 15 and 17 years old (27). However, heroin use in developed countries is increasing (32); during the period of 2000–2012, NAS associated with use of heroin or an opioid prescription increased by fivefold (18). Mothers who abuse heroin are usually unmarried, unemployed, less educated and less insured; which usually implies that the pregnancies are unplanned and have minimal, if any, prenatal care (32).

State differences are noted when analyzing the incidence of NAS. A CDC report that included 28 states found an overall incidence of 6 cases per 1,000 hospital births in 2013 (82). This report also shows a wide difference among states (0.7 per 1,000 vs. 33.4 per 1,000 in Hawaii and West Virginia, respectively) (82). These differences can be attributed to opioid prescribing policies, prevalence of illicit opioid use, or using a different diagnostic code to classify the disease. Variations among states must be taken into consideration when designing public health policies for the prevention of NAS.

Strategies to decrease the incidence of NAS must start during the preconception period with an adequate planning and an honest discussion of how long-term opioid use affects the pregnancy and the infant (31, 82). Prescribed opioids during pregnancy is still one of the most common causes of NAS and should be carefully considered for each case (30). All states should have a prescription drug monitoring program, which has been demonstrated to be satisfactory in reducing inadequate prescribing and overdose deaths (82). Early identification of pregnant women who use illicit drugs is vital in order to improve outcomes (31), however, pregnant women need to have access to comprehensive, fear free treatment options that include medication-assisted treatment, prenatal care and psychosocial support with an honest discussion about the infant’s future (31).

## Conclusion

A national effort is needed to improve maternal health in the prenatal period and to standardize the assessment and management of their infants with NAS (3, 27, 32, 65). Research strategies need to take into consideration that a large number of mothers are not in a supervised maintenance program and are polydrug users (27, 39). Assessment of infants with NAS needs to be standardized (32) and the chosen assessment tool needs to have strong clinimetrics (57) rather than psychometric properties (83). Non-pharmacological approaches need to be offered to all mothers and infants that are affected (32, 69, 84). Infants diagnosed with NAS should be managed, when possible, in a setting where constant rooming-in is available (69, 74).

Recommendations for the pharmacological treatment of NAS, when indicated, need to come from randomized clinical trials with regard to which treatment to initially provide, how to escalate the dose and how to wean the medication(s), taking into consideration the safest time to be discharged home (32, 49). The current lack of education of the many disciplines involved in the assessment and treatment of drug dependence during pregnancy and NAS, makes it difficult for clinicians and researchers to approach this epidemic and to avoid the potential detrimental consequences to this maternal/infant dyad (27, 31, 69, 85). Clinicians, researches, and government funding agencies need to combine their expertise to provide adequate education and treatment protocols for drug dependent pregnant women and their infants with NAS. Without this, they will not live a full and healthy life due to this chronic relapsing disease with the potential to increase the intergenerational transmission of drug dependence and potentiate the epidemic.

## Author Contributions

EG-P conceived the work, drafted and revised the manuscript, approved the final manuscript as submitted, and agreed to be accountable for all aspects of the work. LF assisted in the conception of the work, made critical input and assisted in revisions, approved the final manuscript as submitted, and agreed to be accountable for all aspects of the work.

## Conflict of Interest Statement

The authors declare that the research was conducted in the absence of any commercial or financial relationships that could be construed as a potential conflict of interest.
